# In-Life Testicular Volume as an Indicator of Histological Testicular Development Suitable for Testicular Toxicity Evaluation in Male Cynomolgus Macaques (*Macaca fascicularis*)

**DOI:** 10.3390/ani16162592

**Published:** 2026-08-19

**Authors:** Ryosuke Kawashima, Emiko Haruyama, Akiko Moriyama, Kensuke Mizuyoshi, Atsushi Asano

**Affiliations:** 1 Drug Safety Research Laboratories, Shin Nippon Biomedical Laboratories, Ltd., Kagoshima 891-1394, Japan; 2 Joint Faculty of Veterinary Medicine, Kagoshima University, Kagoshima 890-0065, Japan

**Keywords:** orchidometry, spermatogenesis, puberty, reproductive toxicology, animal selection, nonclinical safety assessment

## Abstract

Reliable evaluation of testicular toxicity in nonclinical safety studies requires animals with sufficient histological testicular maturity. However, chronological age alone is an unreliable indicator of reproductive maturity in cynomolgus macaques because substantial inter-individual variation exists in pubertal development. Since histological maturity can only be confirmed after necropsy, a practical, non-invasive method for prospective animal selection is needed. This retrospective study evaluated whether in-life testicular volume could predict histological maturity suitable for routine histopathological evaluation. Testicular length and width were measured immediately before scheduled necropsy in 61 male cynomolgus macaques (*Macaca fascicularis*) using digital calipers, and testicular volume was calculated using the ellipsoid formula. Histological maturity was classified according to the six-grade system of Haruyama et al., with bilateral Grade ≥4 selected as the primary endpoint because this represents the earliest stage at which routine histopathological evaluation of spermatogenesis is considered reliable. Logistic regression and receiver operating characteristic (ROC) analyses demonstrated a strong association between mean testicular volume and bilateral Grade ≥4 status (AUC = 0.976), with an optimal ROC-derived cut-off value of approximately 8.7 cm^3^. Although age and body weight were significantly associated with histological maturity, testicular volume remained an independent predictor after adjustment for these variables. Testicular width also showed high diagnostic performance, whereas postmortem indices, including testicular weight and the mean relative testicular weight, cannot be applied for prospective animal selection. These findings suggest that externally measured testicular volume may facilitate prospective identification of animals likely to possess testes suitable for routine histopathological evaluation before study initiation.

## Simple Summary

Testicular toxicity is an important endpoint in nonclinical safety studies, but accurate histopathological evaluation requires animals that have reached sufficient testicular maturity. In practice, however, chronological age does not always reflect the developmental status of the testes in cynomolgus macaques because considerable individual variation exists in the timing of sexual maturation. Histological maturity can be determined only after necropsy, making it unsuitable for selecting animals before study initiation. In this study, we investigated whether testicular volume measured non-invasively in living animals could predict histological maturity. Testicular volume showed a strong association with histological Grade 4 or higher, the earliest stage considered suitable for routine histopathological evaluation, and remained an independent predictor after adjustment for age and body weight. Although postmortem indices such as testicular weight and the mean relative testicular weight showed excellent diagnostic performance, they cannot be used for prospective animal selection. These findings suggest that externally measured testicular volume may provide a practical and quantitative method for identifying animals likely to possess sufficiently mature testes before nonclinical safety studies.

## 1. Introduction

Male reproductive toxicity is an important endpoint in nonclinical safety studies because drug-induced testicular injury may impair fertility and reproductive function. Consequently, regulatory guidelines emphasize the importance of evaluating the male reproductive system during general toxicity studies and dedicated reproductive toxicity studies using sexually mature animals whenever appropriate [1,2]. Histopathological examination remains the gold standard for assessing testicular toxicity; however, accurate interpretation requires testes that have reached an appropriate stage of physiological and histological development. Immature testes continue to undergo normal developmental changes, making it difficult to distinguish treatment-related lesions from physiological maturation and potentially confounding the interpretation of toxicological findings [2,3,4,5,6,7]. Therefore, selecting animals with sufficient histological maturity before study initiation is essential for reliable evaluation of male reproductive toxicity.

In cynomolgus macaques (*Macaca fascicularis*), sexual maturity has traditionally been estimated using chronological age, body weight, or external genital development [8,9,10,11,12,13,14]. However, substantial inter-individual variation exists in the timing of puberty, even among animals of similar age and body weight [8,10,11,12,13]. Consequently, animals of the same chronological age may exhibit markedly different stages of testicular development. Because neither chronological age nor body weight reliably reflects histological maturity in individual animals, selecting sexually mature animals solely on the basis of these parameters remains challenging [3,4,11].

Haruyama et al. established a six-grade histological classification system describing postnatal testicular development in cynomolgus macaques [15]. In a companion morphometric study, the same authors demonstrated that growth of the testes and accessory reproductive organs closely parallels histological maturation [16]. According to this classification, Grade 4 represents the earliest developmental stage at which the seminiferous epithelium contains sufficiently differentiated germ-cell populations to permit routine microscopic evaluation of spermatogenesis, whereas Grades 5 and 6 represent progressively mature testes [15,16]. Because histological grading can only be performed after necropsy, however, it cannot be applied prospectively when selecting animals before nonclinical safety studies.

Several methods have been proposed to estimate sexual maturity in living cynomolgus macaques. Testicular weight has been reported to correlate with histological maturation but can only be measured after necropsy [16]. Ku et al. developed a simple orchidometric method based on external measurements to provide a practical preliminary assessment of sexual maturity in male cynomolgus macaques used for nonclinical safety studies [9]. Subsequently, Mirsky et al. demonstrated that orchidometric parameters, including testicular length, width, and calculated volume, are useful indicators of sexual maturation in this species [12]. However, these studies primarily focused on predicting general sexual maturity rather than identifying the minimum histological stage appropriate for routine toxicologic pathology. In humans, ultrasonographic measurement of testicular volume is considered the clinical reference standard because of its high accuracy [17,18,19,20,21,22]. Nevertheless, digital calipers remain attractive for routine laboratory animal use because they are inexpensive, rapid, non-invasive, and require no specialized equipment. Whether externally measured testicular volume can accurately predict the histological maturity required for routine microscopic evaluation in cynomolgus macaques has not been fully investigated.

The objective of the present study was therefore to determine whether in-life testicular volume measured using digital calipers could predict bilateral histological Grade ≥4 in male cynomolgus macaques. In addition, we compared the predictive performance of testicular volume with chronological age, body weight, testicular length, testicular width, and postmortem morphometric indices, and evaluated whether testicular volume remained independently associated with histological maturity after adjustment for age and body weight. The findings of this study may contribute to more objective and reproducible selection of sexually mature cynomolgus macaques for nonclinical safety studies by providing a practical, quantitative, and non-invasive indicator of histological maturity before study initiation.

To our knowledge, no previous study has evaluated whether externally measured testicular volume can predict the minimum histological stage considered suitable for routine toxicologic pathology in male cynomolgus macaques.

## 2. Materials and Methods

### 2.1. Animals and Study Design

The animal study protocol was approved by the Institutional Animal Care and Use Committee of Shin Nippon Biomedical Laboratories, Ltd. (protocol code IACUC999-533, approved on 28 May 2012). This retrospective observational study included 61 purpose-bred male cynomolgus macaques (*Macaca fascicularis*), corresponding to 122 testes. Animals were imported from the Shin Nippon Biomedical Laboratories Cambodia breeding colony and maintained at an AAALAC International fully accredited facility.

The animals ranged in age from 3.6 to 8.5 years (mean ± SD, 5.1 ± 0.9 years) and in body weight from 2.92 to 7.41 kg (mean ± SD, 4.89 ± 1.00 kg). Only animals assigned to control groups in nonclinical toxicity studies were included. All animals underwent scheduled necropsy according to the respective study protocols, and no animals were euthanized specifically for the present study.

All experimental procedures were conducted in accordance with the *Guide for the Care and Use of Laboratory Animals* [23].

Testicular measurements were performed immediately before scheduled necropsy. Therefore, the interval between in-life testicular measurements and tissue collection for histopathological evaluation was 0 days for all animals (mean ± SD, 0.0 ± 0.0 days; range, 0–0 days).

### 2.2. In-Life Testicular Measurements

**Figure 1:**
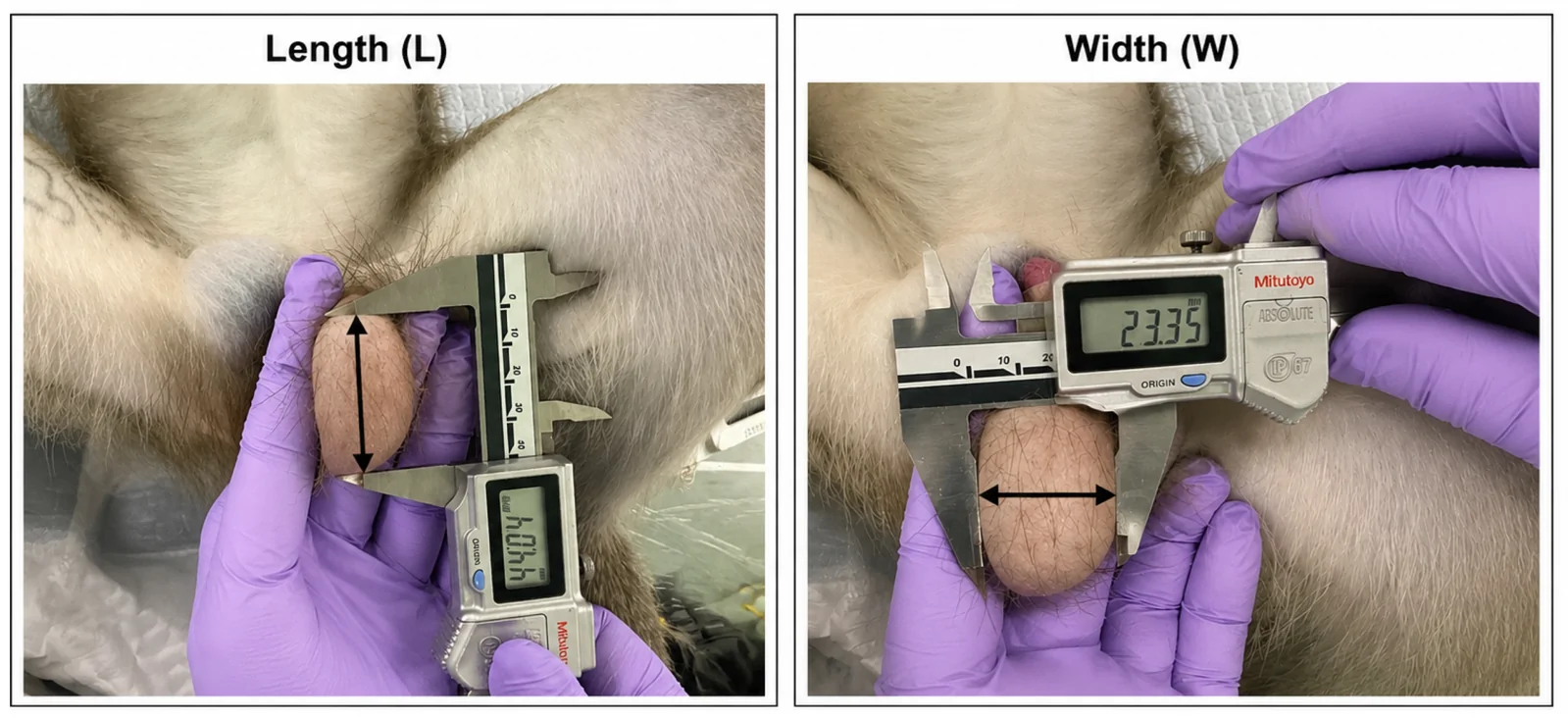
Representative photographs illustrating in-life testicular measurements using digital calipers. Each testis was gently stabilized through the scrotal skin without excessive compression. The maximum longitudinal dimension was measured as length (L), and the maximum transverse dimension of the testicular body was measured as width (W). Care was taken to exclude epididymal tissue from the width measurement.

Immediately before scheduled necropsy, testicular length and width were measured externally under ketamine sedation using a digital caliper (ABS Solar Digimatic Caliper, Mitutoyo Corporation, Kawasaki, Japan). Testicular length was defined as the maximum longitudinal axis of the testis, whereas testicular width was measured at the widest point perpendicular to the longitudinal axis. Care was taken to exclude the epididymis from all measurements (Figure 1). Testicular measurements were performed under ketamine sedation as part of the routine pre-necropsy procedure.

Measurements were performed by one of three trained operators according to a standardized measurement procedure routinely used at SNBL. Because each animal was measured by only one operator and the same animals were not independently measured by multiple operators, formal inter-operator repeatability could not be assessed.

Testicular volume (cm^3^) was calculated using the ellipsoid formula:

Volume = π/6 × Length × Width^2^

Scrotal skin thickness was not subtracted from the measurements because the objective of the present study was to evaluate a practical measurement method applicable under routine laboratory conditions.

### 2.3. Necropsy and Histopathology

Immediately after scheduled necropsy, both testes were excised and processed for histopathological examination according to the procedures previously described by Haruyama et al. [15]. Briefly, testes were fixed, routinely processed, embedded in paraffin, sectioned, and stained with hematoxylin and eosin (H&E).

At necropsy, the left and right testes were separated from the epididymides and surrounding tissues and weighed individually before fixation. For animal-level analyses, mean testicular weight was calculated as the average of the left and right testicular weights. Mean relative testicular weight was calculated by dividing the mean testicular weight (g) by body weight (kg) and was expressed as g/kg.

Histological maturation was evaluated using the six-grade classification system established by Haruyama et al. [15]. This grading system classifies postnatal testicular development according to seminiferous tubular morphology and the progression of spermatogenesis. Representative histological findings for each grade are summarized in Supplementary Table S1, and representative H&E photomicrographs of the testes and epididymides across Grades 1–6 are presented in Supplementary Figure S1 and Figure S2, respectively.

For the primary analysis, bilateral histological Grade ≥4 was defined as histological maturity. Grade 4 was selected because it represents the earliest developmental stage at which seminiferous tubules contain sufficiently differentiated germ-cell populations to permit routine microscopic evaluation of spermatogenesis and histopathological assessment of testicular toxicity [4,15].

Histopathological grading was initially performed according to the criteria described by Haruyama et al. [15]. Approximately 10 representative slides covering the range of developmental grades were subsequently reviewed by a board-certified veterinary pathologist (Akiko Moriyama, DVM, PhD, Japanese College of Veterinary Pathologists), who was blinded to the in-life testicular measurements. Consensus was reached for these representative specimens. Because all slides were not independently evaluated by two observers, formal intra- or inter-observer agreement statistics were not calculated.

For animal-level analyses, animals were classified as histologically mature only when both testes were graded as Grade ≥4. Animals with bilateral Grades 1–3 were classified as histologically immature.

### 2.4. Statistical Analysis

Continuous variables are presented as the median (interquartile range) or mean ± standard deviation (SD), as appropriate. The primary endpoint was bilateral histological maturity, defined as bilateral histological Grade ≥4. Therefore, all primary statistical analyses were performed at the animal level (*n* = 61). Testis-level analyses were additionally performed to evaluate the relationship between histological grade and individual testicular volume.

Differences in age, body weight, mean testicular length, mean testicular width, mean testicular volume, mean testicular weight, and mean relative testicular weight between histologically mature (bilateral Grade ≥4) and immature (bilateral Grades 1–3) animals were evaluated using the Mann–Whitney U test. Associations between continuous animal-level variables were evaluated using Spearman’s rank correlation coefficient. Testis-level data across histological Grades 1–6 were summarized descriptively to illustrate the distribution of testicular volume. Because the left and right testes from the same animal were not statistically independent, no inferential grade-wise comparisons were performed using the 122 individual testes.

To evaluate whether testicular volume was independently associated with histological maturity, binary logistic regression analyses were performed [24] using bilateral histological Grade ≥4 as the dependent variable. Four models were constructed: (1) mean testicular volume alone; (2) mean testicular volume adjusted for chronological age; (3) mean testicular volume adjusted for body weight; and (4) mean testicular volume adjusted for both chronological age and body weight. Regression coefficients (β), odds ratios (ORs), 95% confidence intervals (CIs), and *p* values were calculated for each model. Model performance was evaluated using McFadden’s pseudo-R^2^ [25]. Complete regression coefficients for all models are presented in Supplementary Table S2.

The general model was specified as logit [P(Y = 1)] = β0 + β1(mean testicular volume) + β2(age) + β3(body weight), where Y = 1 indicated bilateral Grade ≥4 status and Y = 0 indicated bilateral Grades 1–3. Terms not included in a given model were omitted according to the model definitions above.

Receiver operating characteristic (ROC) curve analyses were performed to evaluate the ability of chronological age, body weight, mean testicular length, mean testicular width, mean testicular volume, mean testicular weight, and mean relative testicular weight to discriminate animals with bilateral histological Grade ≥4 testes. The area under the ROC curve (AUC), optimal cut-off values determined using the Youden index [26], sensitivity, specificity, and 95% confidence intervals calculated using the DeLong method [27] were obtained. Formal pairwise statistical comparisons between ROC curves were not performed.

All statistical analyses were performed using R version 4.4.2 (R Foundation for Statistical Computing, Vienna, Austria). All statistical tests were two-sided, and *p* values < 0.05 were considered statistically significant.

## 3. Results

### 3.1. Animal Characteristics and Histological Classification

The study included 61 male cynomolgus macaques, corresponding to 122 testes. The animals ranged in age from 3.58 to 8.50 years and in body weight from 2.92 to 7.41 kg. At the animal level, 39 animals were classified as histologically mature (bilateral Grade ≥4), whereas 22 were classified as histologically immature (bilateral Grades 1–3). No animal showed discordant left–right classification across the Grade 4 threshold. Overall animal characteristics and testicular measurements are summarized in Table 1, and the distribution of individual testes across histological Grades 1–6 is shown in Table 2.

Mature animals were older and heavier and had greater testicular dimensions, calculated testicular volume, directly measured testicular weight, and mean relative testicular weight than immature animals (Table 3). Although the age distributions differed significantly, substantial overlap was observed between the maturity groups.

### 3.2. Histopathological Findings and Grade-Dependent Testicular Growth

Histological examination classified the testes into six developmental grades according to the criteria of Haruyama et al. Grades 1 and 2 represented immature and early developing testes, Grade 3 represented the onset and progression of spermatogenesis, Grade 4 represented the minimum stage considered suitable for routine microscopic evaluation, and Grades 5 and 6 represented progressively mature testes. Representative H&E-stained sections of the testes and epididymides are presented in Supplementary Figure S1 and Figure S2, respectively.

**Figure 2:**
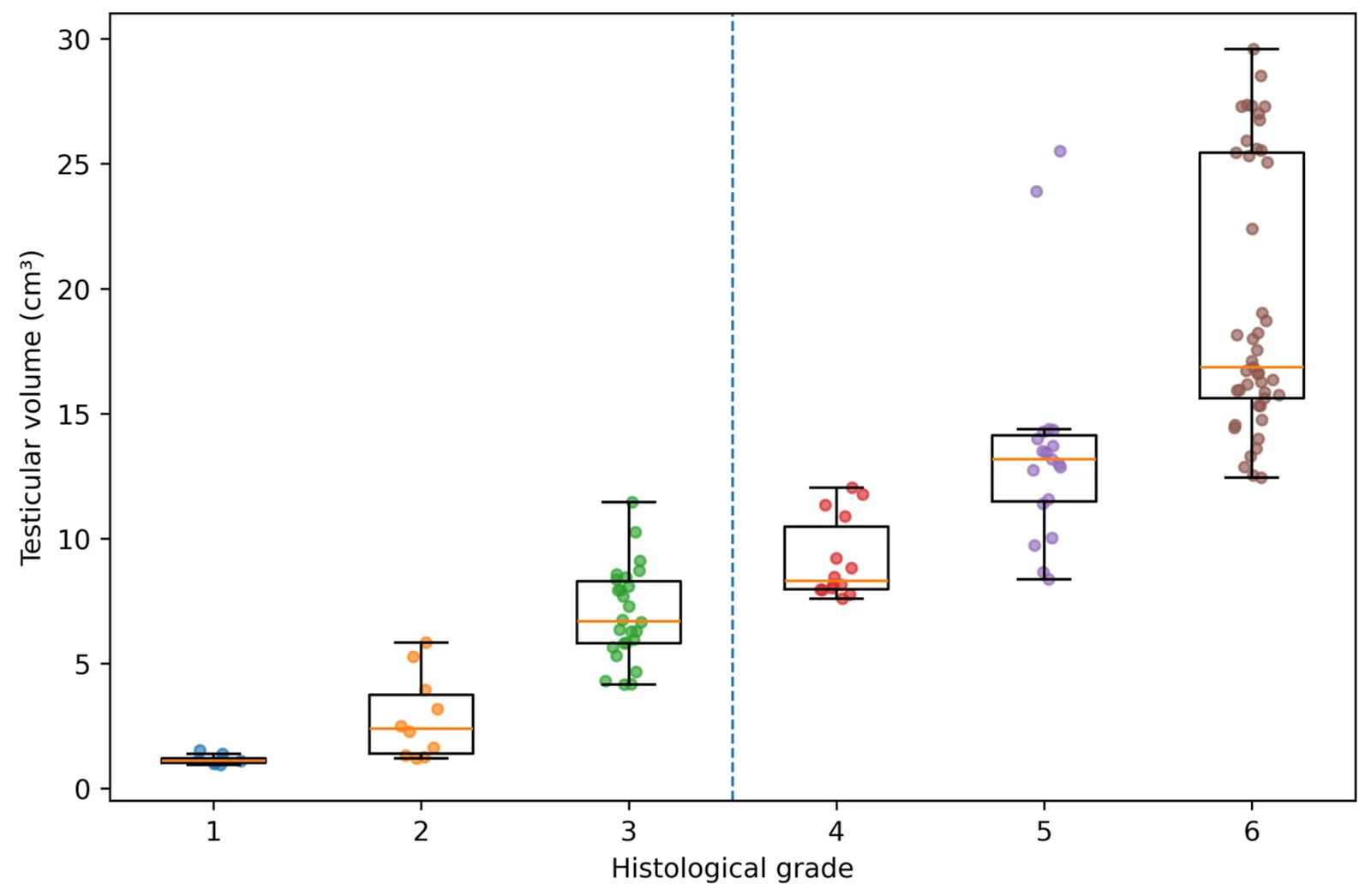
Testicular volume according to histological grade. Each point represents one testis, and horizontal bars indicate median values. The data are presented descriptively because the left and right testes from the same animal are not statistically independent. The dashed vertical line separates Grades 1–3 from Grades 4–6, corresponding to the predefined threshold for histological maturity. Grade 4 was considered the minimum stage sufficiently developed for routine testicular toxicity evaluation.

Testicular volume increased descriptively with advancing histological grade (Table 2 and Figure 2). The clearest separation was observed between Grades 1–2 and Grades 5–6. However, overlap remained between adjacent developmental stages, particularly Grades 3 and 4, indicating that the proposed volume threshold should be interpreted as a probabilistic screening criterion rather than an absolute determinant of histological grade.

### 3.3. Relationships Among Age, Testicular Volume, and Histological Maturity

Animals with bilateral Grade ≥4 testes were older than those with bilateral Grades 1–3 testes (median, 5.50 vs. 4.33 years; Mann–Whitney U test, *p* = 0.00013). However, the age ranges overlapped substantially between the mature and immature groups (3.92–8.50 and 3.58–6.75 years, respectively).

Mean testicular volume was positively correlated with age (Spearman’s ρ = 0.539, *p* < 0.001). Nevertheless, the overlap in age across histological categories indicated that chronological age alone could not accurately determine individual histological testicular maturity.

### 3.4. Logistic Regression Analysis

**Figure 3:**
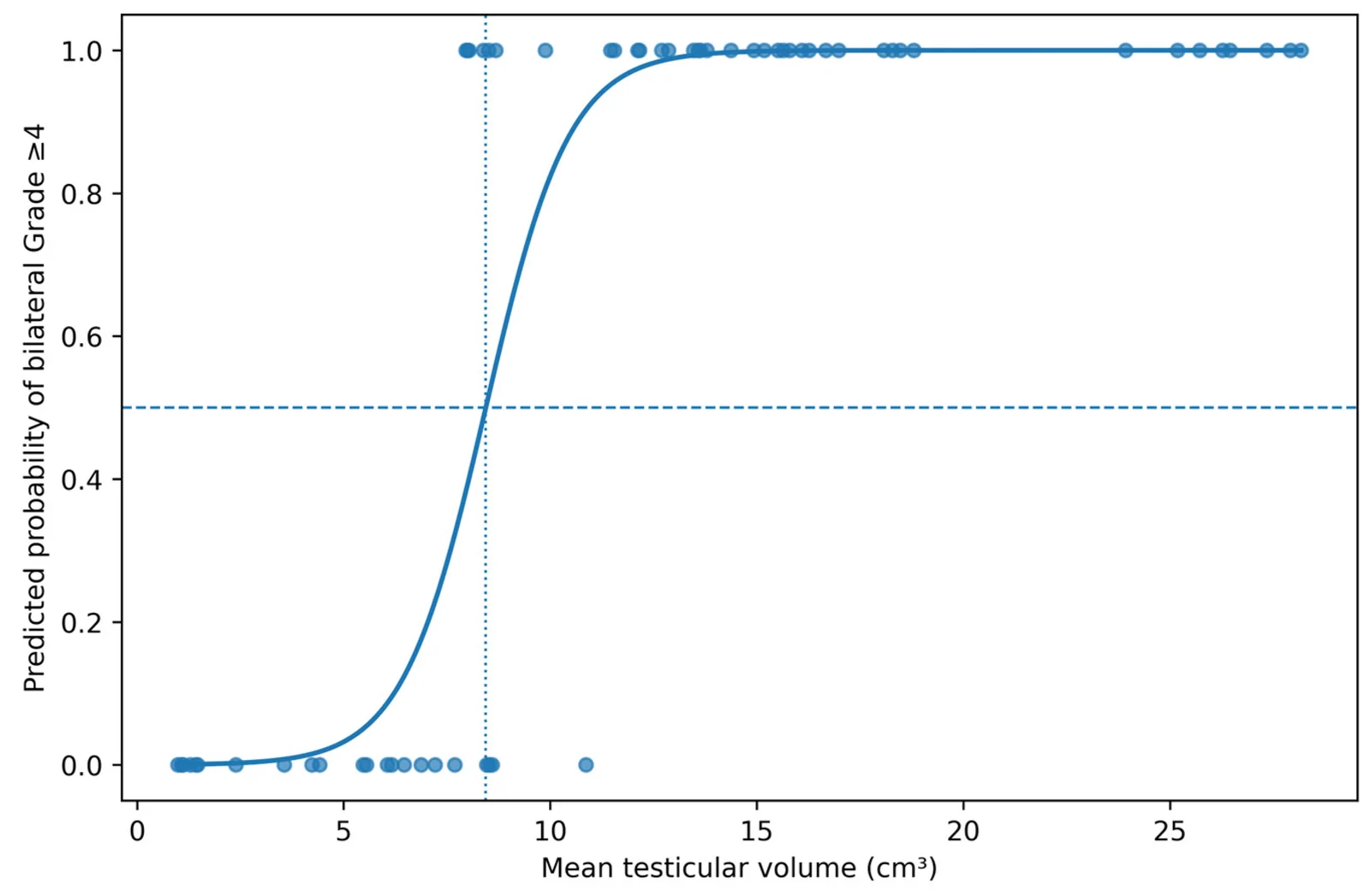
Animal-level logistic regression model showing the relationship between the mean bilateral testicular volume and the predicted probability of bilateral Grade ≥4 status. Mean bilateral testicular volume was calculated as the average of the left and right testicular volumes for each animal. Each point represents one animal, classified as bilateral Grades 1–3 or bilateral Grade ≥4. The solid curve represents the predicted probability estimated from the volume-only logistic regression model. Bilateral Grade ≥4 status was defined as both testes having reached at least the minimum histological stage considered suitable for testicular toxicity evaluation. The horizontal and vertical dashed lines indicate a predicted probability of 0.5 and the corresponding mean bilateral testicular volume threshold, respectively.

The relationship between mean testicular volume and the predicted probability of bilateral Grade ≥4 status is illustrated in Figure 3. In the univariate animal-level model, mean testicular volume was significantly associated with bilateral Grade ≥4 status. This association remained significant after separate adjustment for age and body weight and after simultaneous adjustment for both covariates (Table 4). Neither age nor body weight was independently significant in the adjusted models. McFadden’s pseudo-R^2^ increased from 0.719 in the volume-only model to 0.771 in the model including volume, age, and body weight.

### 3.5. Receiver Operating Characteristic Analysis

**Figure 4:**
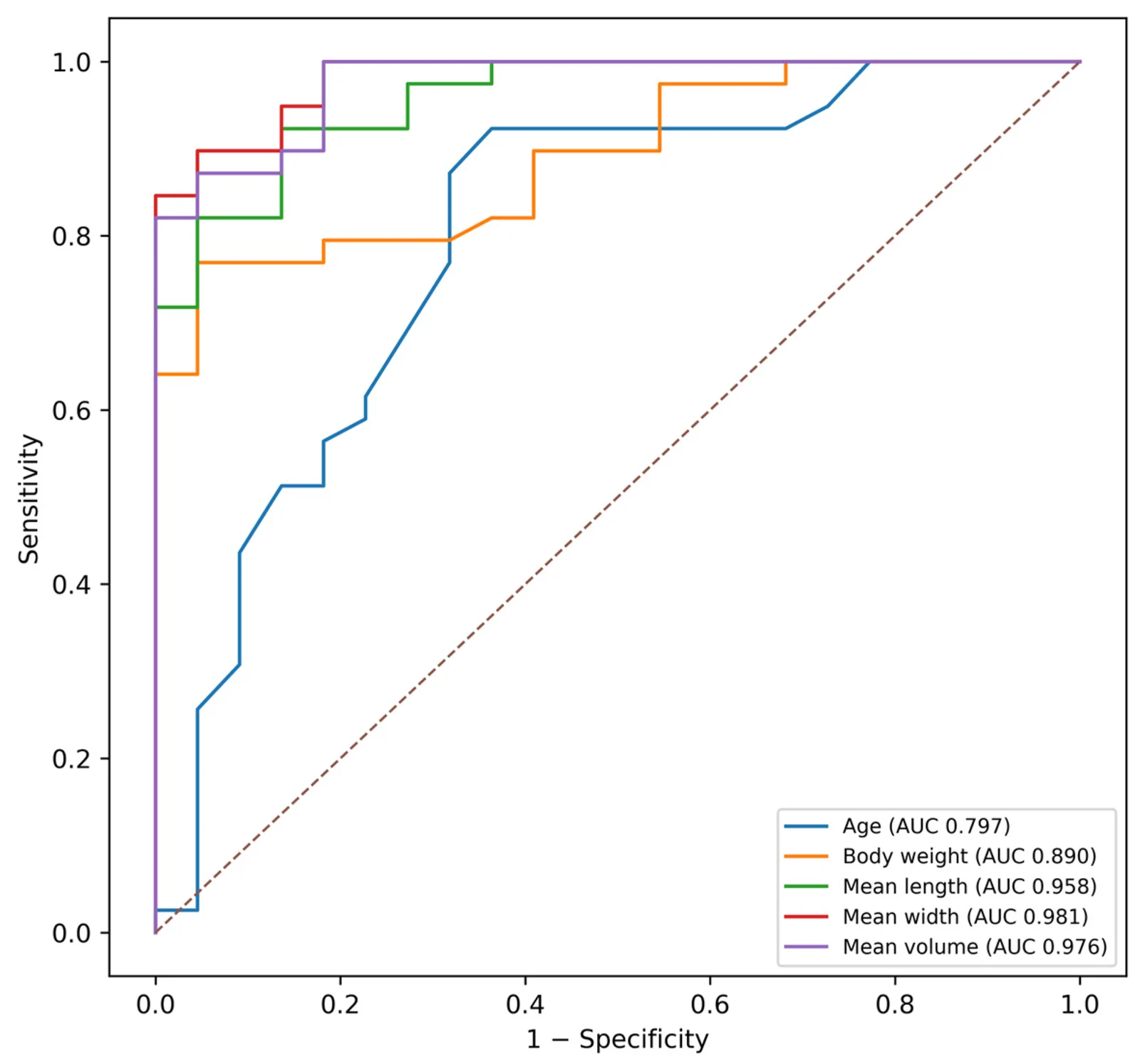
Receiver operating characteristic curves for parameters available before study initiation for discrimination of bilateral Grade ≥4 status at the animal level. Curves are shown for age, body weight, mean bilateral testicular length, mean bilateral testicular width, and mean bilateral testicular volume. Mean bilateral measurements were calculated as the average of the left and right testes for each animal. Postmortem mean testicular weight and mean relative testicular weight were not included because these parameters are unavailable for prospective animal selection. No formal pairwise comparison of AUCs was performed.

Among parameters available before study initiation, mean testicular width showed the highest AUC (0.981), followed by mean testicular volume (0.976) and mean testicular length (0.958). Body weight showed moderate-to-high discrimination (AUC, 0.890), whereas age showed lower discriminatory performance (AUC, 0.797) (Table 5 and Figure 4). The optimal cut-off for mean testicular volume was 8.68 cm^3^, with a sensitivity of 87.2% and specificity of 95.5%.

Postmortem mean testicular weight and mean relative testicular weight yielded AUCs of 0.997 and 0.991, respectively. Although these indices showed excellent discrimination, neither can be used for prospective animal selection because both require necropsy-derived measurements.

### 3.6. Overall Predictive Performance

Collectively, the analyses showed that testicular dimensions measured in life had numerically higher AUCs than chronological age or body weight. Mean testicular volume remained independently associated with bilateral Grade ≥4 status after adjustment for both age and body weight. Mean testicular width and calculated testicular volume showed comparably high discriminatory performance. Width may represent a simpler alternative for rapid screening, whereas calculated volume integrates longitudinal and transverse testicular growth and facilitates comparison with previous volumetric studies.

## 4. Discussion

The present study demonstrated that in-life testicular dimensions measured using digital calipers were strongly associated with histological testicular maturity in male cynomolgus macaques. Mean testicular volume showed excellent discriminatory performance for identifying animals with bilateral histological Grade ≥4 and remained significantly associated with maturity after adjustment for chronological age and body weight. These findings indicate that testicular volume provides information on testicular development beyond general somatic growth and may serve as a practical screening parameter before animals are assigned to nonclinical safety studies.

The histological endpoint used in this study was based on the six-grade classification established by Haruyama et al. [15]. Grade 4 was selected as the primary threshold because it represents the earliest stage at which seminiferous tubules contain the major germ-cell populations necessary for routine evaluation of spermatogenesis. Grades 5 and 6 represent progressively more mature testes, whereas Grades 1–3 are characterized by incomplete testicular development [15,16]. The purpose of the present study was not to redefine sexual maturity, but to determine whether this previously established histological stage could be predicted using a non-invasive in-life measurement.

This distinction is important because Grade 4 should not necessarily be considered equivalent to full adult maturity. Physiological development may still be ongoing at this stage, and the present study did not directly assess whether Grade 4 testes respond to reproductive toxicants in the same manner or with the same sensitivity as Grade 5 or 6 testes. Therefore, the proposed threshold should be interpreted as identifying the minimum histological stage considered suitable for routine microscopic evaluation rather than proving complete reproductive maturity or equivalent toxicant sensitivity. Prospective studies using known testicular toxicants will be required to determine whether Grade 4 animals provide sensitivity comparable with that of fully mature animals.

Chronological age was significantly associated with both histological maturity status and testicular volume. However, considerable overlap in age was observed between mature and immature animals. Some animals classified as Grade ≥4 were younger than animals remaining at Grades 1–3, indicating that chronological age alone cannot reliably determine individual testicular maturity. This is consistent with previous reports showing substantial inter-individual variation in pubertal development in cynomolgus macaques [3,8,10,11,12,13,14]. In the age-adjusted logistic regression model, testicular volume remained significantly associated with Grade ≥4, whereas age was not independently significant. These findings suggest that direct assessment of testicular development provides information beyond chronological age alone.

Body weight also differed between mature and immature groups and showed moderate discriminatory performance in ROC analysis. Nevertheless, mean testicular volume remained significantly associated with Grade ≥4 after adjustment for body weight and after simultaneous adjustment for age and body weight. Body weight reflects overall somatic growth and may be influenced by nutritional status, body composition, and colony management conditions. Consequently, it may not accurately represent reproductive-organ development in individual animals. The multivariable results indicate that testicular volume is not merely a surrogate for larger or older animals, but a more direct indicator of testicular development.

Direct measurements of testicular length and width also demonstrated high discriminatory performance. Mean testicular width and calculated testicular volume showed comparably high AUCs (0.981 and 0.976, respectively). Because no formal pairwise comparison of AUCs was performed, neither parameter can be considered statistically superior. Testicular width may therefore represent a simpler alternative for rapid preliminary screening, whereas calculated volume integrates both longitudinal and transverse growth and facilitates comparison with previous orchidometric, ultrasonographic, and volumetric studies [9,12,17,18,19,20,21,22]. Accordingly, width and volume should both be regarded as high-performing practical morphometric indicators.

The optimal cut-off value for mean testicular volume was approximately 8.7 cm^3^ in the present animal-level analysis. This ROC-derived cut-off is close to the 50% predicted-probability threshold of approximately 8.5 cm^3^ estimated from the volume-only logistic regression model and corresponds to a bilateral total volume of approximately 17 cm^3^. However, substantial overlap was observed between Grades 3 and 4, and some Grade 5 testes also had volumes near the cut-off. Measurements close to the cut-off should therefore be interpreted cautiously and, where feasible, considered together with additional information such as age, body weight, serial measurements, semen evaluation, hormonal assessment, or ultrasonography. The cut-off should not be interpreted as an absolute biological boundary; rather, it represents a data-derived probabilistic screening threshold that maximized sensitivity and specificity in the present cohort. Its performance should be externally validated before adoption as a universal criterion across breeding colonies, age distributions, or facilities.

Postmortem mean testicular weight and mean relative testicular weight showed excellent discriminatory performance and numerically exceeded the in-life parameters. These findings are biologically plausible because testicular weight directly reflects testicular mass, whereas relative testicular weight adjusts testicular mass for body size. However, neither measure can be obtained before study allocation because both require necropsy-derived measurements. Their value in the present study is therefore primarily as postmortem biological benchmarks supporting the relationship between testicular size and histological development.

The caliper method also offers practical advantages over ultrasonography. Ultrasonography can provide more anatomically precise measurements and avoid inclusion of scrotal tissue, but it requires specialized equipment, operator expertise, and additional procedure time [17,18,19,20,21,22]. Digital calipers are inexpensive, portable, and readily available in most laboratory animal facilities. In the present study, measurements were made through the scrotal skin, and skin thickness was not subtracted. This may have introduced a small systematic overestimation of true testicular dimensions. Nevertheless, the objective was to evaluate a method that could be applied consistently under routine laboratory conditions. The high discriminatory performance observed despite this limitation supports the practical utility of the approach.

The present study may also contribute to refinement in animal use. Selecting animals with a high probability of adequate histological maturity before study initiation may reduce the inclusion of immature animals whose normal developmental changes could complicate interpretation. This could improve study reliability, reduce avoidable variability, and decrease the need to repeat studies because of unsuitable reproductive maturation status. However, the method should supplement rather than replace comprehensive animal selection criteria, including age, body weight, clinical condition, reproductive history, and study-specific requirements.

Several limitations should be acknowledged. First, this was a retrospective study using animals from a single facility and a single breeding-colony background. External validation in independent cohorts is required. Second, caliper-derived volume was not validated against ultrasonography or another imaging method. Third, each animal was measured by only one of three trained operators; because the same animals were not independently measured by multiple operators, formal inter-operator repeatability could not be assessed. Fourth, formal intra- and inter-observer reproducibility of histological grading was not evaluated. The initial grading was performed by the author, and approximately 10 representative slides were reviewed by a board-certified veterinary pathologist, with agreement confirmed. However, this does not constitute a complete blinded double-reading study or permit calculation of a weighted κ statistic. Fifth, no formal pairwise statistical comparison of ROC curves was performed. Finally, the present study evaluated association with histological maturity but did not directly test reproductive function, fertility, endocrine status, or sensitivity to testicular toxicants. The wide confidence intervals for body weight in the multivariable models indicate limited precision, likely reflecting the modest sample size and correlations among age, body weight, and testicular dimensions.

## 5. Conclusions

In conclusion, in-life testicular volume measured using digital calipers was strongly associated with histological testicular maturity in male cynomolgus macaques and remained independently associated with bilateral histological Grade ≥4 after adjustment for chronological age and body weight. Compared with chronological age or body weight alone, testicular volume showed a numerically higher AUC for discriminating histological developmental status and therefore represents a practical, non-invasive parameter for prospective animal selection.

Although testicular width also demonstrated excellent discriminatory performance, calculated testicular volume integrates multiple dimensions of testicular growth and provides a standardized metric that is readily comparable with previous studies. The proposed cut-off value should be regarded as a practical screening threshold rather than an absolute criterion for sexual maturity and should be validated in independent populations before routine implementation.

Overall, caliper-based testicular volume measurement offers a simple, inexpensive, and readily implementable approach for identifying male cynomolgus macaques likely to possess testes suitable for routine histopathological evaluation in nonclinical safety studies. Its application may improve animal selection, reduce biological variability, and enhance the reliability and interpretability of reproductive toxicity assessments.

## Figures and Tables

**Table 1:** Study population and testicular measurement summary.

Variable	Value
Animals	61
Testes	122
Age, years, mean ± SD (range)	5.1 ± 0.9 (3.6–8.5)
Body weight, kg, mean ± SD (range)	4.89 ± 1.00 (2.92–7.41)
Testicular volume per testis, cm^3^, mean ± SD (range)	12.11 ± 7.48 (0.94–29.59)
Testicular weight per testis, g, mean ± SD (range)	12.8 ± 7.8 (0.9–34.1)

Age and body weight are summarized at the animal level; testicular volume and testicular weight are summarized at the individual-testis level.

**Table 2:** Distribution of histological maturity grades and testicular volume.

Grade	Interpretation	Number of Testes	Mean Volume ± SD (cm^3^)	Median Volume (cm^3^)	Range (cm^3^)
1	Immature	8	1.16 ± 0.20	1.11	0.94–1.52
2	Pre-puberty	10	2.84 ± 1.69	2.39	1.21–5.84
3	Onset of puberty	26	7.00 ± 1.86	6.70	4.16–11.46
4	Puberty	14	9.15 ± 1.62	8.30	7.60–12.04
5	Early adult	18	12.95 ± 3.35	13.08	8.37–23.89
6	Adult	46	19.49 ± 5.37	16.98	12.44–29.59

Data are presented descriptively at the individual-testis level. No inferential grade-wise comparisons were performed because paired testes from the same animal were not statistically independent. Inferential analyses of bilateral Grade ≥4 status were performed at the animal level using the mean values of the left and right testes.

**Table 3:** Comparison of animal-level characteristics between histologically immature and mature groups.

Variable	Grades 1–3 (*n* = 22)	Grade ≥4 (*n* = 39)	*p* Value
Age (years)	4.33 (3.94–4.98)	5.50 (4.92–5.88)	0.0001
Body weight (kg)	4.34 (3.56–4.55)	5.28 (4.80–5.72)	<0.0001
Mean testicular length (mm)	28.37 (22.02–32.47)	39.59 (36.27–43.13)	<0.0001
Mean testicular width (mm)	18.91 (12.00–20.45)	27.22 (24.87–28.92)	<0.0001
Mean testicular volume (cm^3^)	5.51 (1.69–7.13)	15.18 (12.14–18.38)	<0.0001
Mean testicular weight (g)	5.88 (1.77–6.78)	16.20 (12.77–20.23)	<0.0001
Mean relative testicular weight (g/kg)	1.29 (0.55–1.53)	3.18 (2.56–3.79)	<0.0001

Values are median (interquartile range). *p* values were obtained using the Mann–Whitney U test.

**Table 4:** Animal-level logistic regression models predicting bilateral Grade ≥4 status.

Model	Predictor	OR	95% CI	*p* Value	McFadden Pseudo-R^2^
Model 1	Mean testicular volume (per 1 cm^3^)	2.69	1.34–5.40	0.0055	0.719
Model 2	Mean testicular volume (per 1 cm^3^)	2.58	1.34–4.97	0.0048	—
Model 2	Age (per 1 year)	2.33	0.48–11.18	0.2911	0.735
Model 3	Mean testicular volume (per 1 cm^3^)	2.71	1.31–5.58	0.0069	—
Model 3	Body weight (per 1 kg)	18.58	0.54–634.58	0.1048	0.764
Model 4	Mean testicular volume (per 1 cm^3^)	2.68	1.32–5.45	0.0065	—
Model 4	Age (per 1 year)	1.80	0.36–9.11	0.4748	—
Model 4	Body weight (per 1 kg)	13.51	0.38–475.91	0.1520	0.771

Model 1 included mean testicular volume only. Model 2 included mean testicular volume and age. Model 3 included mean testicular volume and body weight. Model 4 included mean testicular volume, age, and body weight. The dependent variable was bilateral histological maturity status, coded as 1 for bilateral Grade ≥4 and 0 for bilateral Grades 1–3.

**Table 5:** ROC performance for discrimination of bilateral Grade ≥4 status.

Parameter	AUC	95% CI	Cut-off	Sensitivity	Specificity
Age	0.797	0.670–0.923	4.67 years	92.3%	63.6%
Body weight	0.890	0.812–0.968	4.78 kg	76.9%	95.5%
Mean length	0.958	0.916–1.000	33.20 mm	92.3%	86.4%
Mean width	0.981	0.957–1.000	22.27 mm	89.7%	95.5%
Mean volume	0.976	0.946–1.000	8.68 cm^3^	87.2%	95.5%
Mean testicular weight	0.997	0.991–1.000	9.70 g	94.9%	100.0%
Mean relative testicular weight	0.991	0.976–1.000	2.06 g/kg	94.9%	95.5%

AUC confidence intervals were calculated using the DeLong method. Cut-offs were selected using the Youden index.

## Data Availability

The data supporting the findings of this study are provided in Supplementary Table S3. Additional information is available from the corresponding author upon reasonable request, where appropriate.

## Supplementary Materials

The following supporting information can be downloaded at https://www.mdpi.com/article/10.3390/ani16162592/s1. Table S1: Histological characteristics of the testes and epididymides across developmental Grades 1–6 in male cynomolgus macaques; Table S2: Complete logistic regression coefficients for Models 1–4; Table S3: Individual animal-level and testis-level measurement and histopathology dataset; Figure S1: Representative histological features of testes across developmental Grades 1–6 in male cynomolgus macaques; Figure S2: Representative histological features of epididymides across testicular developmental Grades 1–6 in male cynomolgus macaques.
