# Supplementary material for: In-Life Testicular Volume as an Indicator of Histological Testicular Development Suitable for Testicular Toxicity Evaluation in Male Cynomolgus Macaques (Macaca fascicularis)
Source: Animals (Basel). 2026 Aug 19;16(16):2592. doi: 10.3390/ani16162592 (PMC13508964; doi:10.3390/ani16162592)
Supplement: Supplementary file 1 [file animals-16-02592-s001.zip › Supplementary Table S1. Histological characteristics of the testes and epididymides across developmental Grades 1–6 in male cynomolgus macaques..PDF]

Supplementary Table S1. Histological characteristics of the testes and epididymides across developmental Grades 1–6 in male cynomolgus macaques.

Grade	Developmental stage	Testicular findings	Epididymal findings	Suitability for testicular toxicity evaluation
1	Immature	Sertoli cells and spermatogonia only; no lumen	No sperm; very slight debris	Not suitable
2	Prepuberty	Round spermatids and early lumen formation; no elongated spermatids	No sperm; very slight debris	Not suitable
3	Onset of puberty	All major germ-cell classes present; small tubules; immature organization/desquamation	Very few to few sperm; moderate debris	Not suitable
4	Puberty	Almost complete spermatogenesis; spermatogenic stages identifiable	Very few to moderate sperm; less debris than Grade 3	Minimum evaluable stage
5	Early adult	Complete spermatogenesis	Moderate to large amounts of sperm	Suitable
6	Adult	Complete spermatogenesis; mature seminiferous tubules	Large to very large amounts of sperm	Suitable
