# Supplementary material for: In-Life Testicular Volume as an Indicator of Histological Testicular Development Suitable for Testicular Toxicity Evaluation in Male Cynomolgus Macaques (Macaca fascicularis)
Source: Animals (Basel). 2026 Aug 19;16(16):2592. doi: 10.3390/ani16162592 (PMC13508964; doi:10.3390/ani16162592)
Supplement: Supplementary file 1 [file animals-16-02592-s001.zip › Supplementary Table S3. Individual animal-level and testis-level measurement and histopathology dataset.pdf]

Supplementary Table S3. Individual animal-level and testis-level measurement and histopathology dataset.

Animal ID	Date of birth	Age at necropsy (months)	Age at necropsy (years)	Body weight (kg)	Left testis length (mm)	Left testis width (mm)	Right testis length (mm)	Right testis width (mm)	Left testicular volume (cm^3)	Right testicular volume (cm^3)	Mean testicular volume (cm^3)	Left absolute testicular weight (g)	Right absolute testicular weight (g)	Left relative testicular weight (g/kg)	Right relative testicular weight (g/kg)	Left testis histopathological grade	Right testis histopathological grade	Left epididymis histopathological grade	Right epididymis histopathological grade
X0811435	2008-11-14	44	3.67	3.81	19.48	10.62	20.17	9.67	1.15	0.99	1.07	1.20	1.20	0.31	0.31	1	1	1	1
W0810221	2008-10-22	45	3.75	3.15	20.07	11.20	19.46	11.10	1.32	1.25	1.29	0.90	0.90	0.29	0.29	2	2	2	2
W0902261	2008-07-11	48	4.00	3.54	21.27	10.44	20.31	12.39	1.21	1.63	1.42	1.00	1.00	0.28	0.28	2	2	2	2
K120313	2007-09-08	58	4.83	4.78	32.93	21.47	33.48	22.43	7.94	8.82	8.38	8.00	8.00	1.67	1.67	4	4	4	4
K120409	2006-11-21	68	5.67	5.94	40.30	29.21	40.26	29.35	17.99	18.15	18.07	20.80	22.40	3.50	3.77	6	6	6	6
K120411	2006-10-12	70	5.83	4.75	28.63	19.92	28.27	19.03	5.95	6.36	6.16	6.40	6.50	1.35	1.37	3	3	3	3
K120469	2007-02-02	66	5.50	4.56	27.86	16.92	26.93	18.18	4.17	4.66	4.42	5.50	5.70	1.21	1.25	3	3	3	3
K120473	2007-04-15	64	5.33	5.37	37.72	27.15	37.48	27.94	14.55	15.31	14.93	16.30	16.10	3.04	3	6	6	6	6
K120507	2009-01-08	43	3.58	4.36	27.52	19.13	29.04	19.60	5.27	5.84	5.55	5.70	7.90	1.31	1.81	2	2	2	2
X0604685	2006-04-21	73	6.08	4.83	41.20	24.83	36.54	25.51	13.29	12.44	12.86	19.10	18.60	3.95	3.85	6	6	6	6
SC0709009	2007-09-10	56	4.67	5.00	33.45	22.81	32.78	21.50	9.11	7.93	8.52	7.40	7.30	1.48	1.46	3	3	3	3
SC0707071	2007-07-24	58	4.83	5.72	34.62	26.30	35.67	26.26	12.53	12.87	12.70	17.70	18.10	3.09	3.16	6	6	6	6
X0706627	2007-06-19	59	4.92	4.54	36.07	23.30	37.61	24.13	10.25	11.46	10.86	8.60	9.20	1.89	2.03	3	3	3	3
SC0708047	2007-08-25	58	4.83	5.71	40.80	27.15	41.41	27.04	15.74	15.85	15.79	17.90	18.50	3.13	3.24	6	6	6	6
061691C	2006-10-11	68	5.67	5.67	43.05	27.36	42.86	27.61	16.86	17.10	16.98	21.40	20.80	3.77	3.67	6	6	6	6
SC0707041	2007-07-14	59	4.92	4.43	49.48	31.10	49.56	31.24	25.05	25.31	25.18	26.90	26.40	6.07	5.96	6	6	6	6
X0611321	2006-11-10	67	5.58	6.25	45.23	28.12	41.38	29.01	18.72	18.22	18.47	18.80	17.20	3.01	2.75	6	6	6	6
SC0707001	2007-07-01	60	5.00	4.52	31.68	21.99	31.87	21.84	8.02	7.96	7.99	9.40	9.50	2.08	2.1	4	4	4	4
W0705181	2007-10-11	56	4.67	4.11	40.25	27.52	40.11	27.02	15.95	15.33	15.64	17.20	17.10	4.18	4.16	6	6	6	6
X0901723	2009-01-23	44	3.67	3.04	24.31	13.39	25.03	13.81	2.28	2.50	2.39	1.50	1.80	0.49	0.59	2	2	2	2
X0901403	2009-01-13	44	3.67	3.32	19.83	9.51	20.41	9.87	0.94	1.04	0.99	0.90	0.90	0.27	0.27	1	1	1	1
X0810265	2008-10-09	47	3.92	2.92	26.49	16.86	25.72	15.38	3.94	3.18	3.56	3.50	3.50	1.20	1.2	2	2	2	2
X0706321	2007-06-10	60	5.00	6.15	33.93	23.41	32.91	24.13	9.73	10.03	9.88	16.30	16.90	2.65	2.75	5	5	5	5
X0603825	2006-03-26	75	6.25	5.21	32.14	22.31	32.97	22.39	8.37	8.65	8.51	11.70	11.70	2.25	2.25	5	5	5	5
X0701539	2007-01-17	67	5.58	6.67	41.37	29.64	38.78	29.40	19.02	17.54	18.28	15.00	16.40	2.25	2.46	6	6	6	6
X0606339	2006-06-11	74	6.17	5.93	35.60	26.86	34.36	27.40	13.44	13.50	13.47	11.90	12.60	2.01	2.12	5	5	5	6
X0608853	2006-08-27	72	6.00	5.72	49.62	33.14	47.88	33.00	28.52	27.29	27.91	24.70	24.50	4.32	4.28	6	6	6	6
X0608889	2006-08-28	70	5.83	5.98	41.77	27.20	42.67	27.06	16.17	16.35	16.26	18.60	19.70	3.11	3.29	6	6	6	6
SC3926	2004-01-06	102	8.50	7.39	50.77	33.37	51.58	31.48	29.59	26.75	28.17	34.10	30.00	4.61	4.06	6	6	6	6
X0806603	2008-06-19	48	4.00	4.45	40.97	27.26	42.10	27.17	15.93	16.26	16.09	15.50	15.30	3.48	3.44	6	6	6	6
X0804277	2008-04-09	50	4.17	4.52	33.58	21.45	32.59	20.66	8.09	7.28	7.69	6.20	6.50	1.37	1.44	3	3	3	3
X0804845	2008-04-26	50	4.17	4.42	32.51	19.90	32.67	21.21	6.74	7.69	7.21	6.40	7.00	1.45	1.58	3	3	3	3
X0803331	2008-03-11	54	4.50	4.77	32.08	19.90	31.68	19.47	6.65	6.28	6.46	5.70	5.20	1.19	1.09	3	3	3	3
X0703931	2007-03-29	66	5.50	5.21	32.37	21.93	32.10	21.51	8.15	7.77	7.96	9.40	10.00	1.80	1.92	4	4	4	4
X0703843	2007-03-27	66	5.50	4.49	33.88	21.84	33.25	20.90	8.46	7.60	8.03	9.50	10.50	2.12	2.34	4	4	4	4
X0609723	2006-09-22	69	5.75	4.87	33.64	21.52	33.23	23.01	8.15	9.21	8.68	9.40	13.10	1.93	2.69	4	4	4	4
X0703877	2007-03-28	63	5.25	4.66	28.88	16.60	26.98	17.46	4.16	4.30	4.23	5.20	5.60	1.12	1.2	3	3	3	3
X0709317	2007-09-10	58	4.83	5.42	42.71	27.28	42.66	27.37	16.63	16.72	16.67	15.40	15.20	2.84	2.8	6	6	6	6
X0707513	2007-07-16	60	5.00	5.02	38.24	25.65	35.56	27.42	13.17	13.99	13.58	13.30	13.90	2.65	2.77	5	5	5	5
X0808757	2008-08-24	47	3.92	5.28	37.66	25.42	36.81	24.51	12.74	11.57	12.16	12.50	11.50	2.37	2.18	5	5	5	5
X0807969	2008-07-31	47	3.92	3.64	39.51	26.01	39.67	25.59	13.99	13.60	13.79	13.30	12.70	3.65	3.49	6	6	6	6
K120135	2007-12-16	55	4.58	4.56	33.30	21.34	30.91	18.95	7.94	5.81	6.88	7.30	7.80	1.60	1.71	3	3	3	3
K111227	2006-02-03	78	6.50	5.05	37.75	25.51	37.71	24.02	12.86	11.39	12.12	12.70	12.60	2.51	2.5	5	5	5	5
K110989	2007-12-04	54	4.50	3.34	18.79	10.69	18.08	10.76	1.12	1.10	1.11	1.60	1.60	0.48	0.48	1	1	1	1
K111075	2008-05-07	49	4.08	3.63	21.27	11.68	20.99	11.24	1.52	1.39	1.46	2.20	2.10	0.61	0.58	1	1	1	1
K111215	2006-07-19	71	5.92	4.18	37.28	24.84	35.91	24.07	12.04	10.89	11.46	10.80	12.10	2.58	2.89	4	4	4	4
K110805	2005-09-27	81	6.75	4.50	34.57	21.76	33.89	21.71	8.57	8.36	8.46	9.60	9.30	2.13	2.07	3	3	3	3
X0511585	2005-11-18	79	6.58	7.41	48.57	31.69	49.09	32.42	25.53	27.00	26.27	23.70	23.70	3.20	3.51	6	6	6	6
X0612819	2006-12-26	66	5.50	5.41	48.45	31.97	48.51	31.70	25.92	25.51	25.72	24.00	24.00	4.44	4.44	6	5	6	5
X0608657	2006-08-21	70	5.83	7.11	46.61	30.30	45.32	32.76	22.39	25.45	23.92	26.20	26.20	3.68	4.05	6	6	6	6
X0703943	2007-03-30	63	5.25	5.72	45.72	33.79	44.14	32.68	27.32	27.35	27.34	32.10	32.10	5.61	5.61	6	6	6	6
K120193	2008-05-28	49	4.08	3.99	31.42	19.56	31.18	18.90	6.29	5.83	6.06	6.20	6.10	1.55	1.53	3	3	3	3
K120573	2007-11-05	56	4.67	5.09	42.53	25.32	40.85	24.65	14.27	12.99	13.63	16.60	15.80	3.26	3.1	5	5	5	5
K120403	2007-02-03	65	5.42	4.32	30.27	18.87	30.22	18.33	5.64	5.31	5.47	6.50	6.50	1.50	1.5	3	3	3	3
K120331	2007-04-15	61	5.08	4.95	35.58	27.76	36.29	27.52	14.35	14.38	14.37	13.40	12.40	2.71	2.51	5	5	5	5
K120405	2007-02-02	64	5.33	4.91	39.94	25.61	47.41	31.03	13.71	23.89	18.80	13.60	25.10	2.77	5.11	5	5	5	5
K120489	2007-07-12	59	4.92	4.59	38.51	27.05	38.95	27.68	14.75	15.62	15.18	15.50	15.90	3.38	3.46	6	6	6	6
K120575	2006-01-15	76	6.33	5.65	38.51	28.69	35.96	27.69	16.59	14.43	15.51	16.70	15.90	2.96	2.81	6	6	6	6
K111303	2005-10-20	80	6.67	5.70	48.14	31.88	48.11	32.92	25.60	27.29	26.45	24.40	23.90	4.28	4.19	6	6	6	6
K120303	2007-08-02	59	4.92	4.29	37.92	23.90	38.12	24.29	11.34	11.77	11.55	13.20	16.10	3.08	3.75	4	4	4	4
K120301	2007-07-12	60	5.00	4.06	34.01	21.79	33.70	22.24	8.45	8.72	8.59	7.60	7.50	1.87	1.85	3	3	3	3

Notes

Testicular volume was calculated using the ellipsoid formula: volume = $(\pi/6) \times \text{length} \times \text{width}^2$.

Testicular weights were measured after removal of the epididymides and surrounding tissues.
