# Supplementary material for: In-Life Testicular Volume as an Indicator of Histological Testicular Development Suitable for Testicular Toxicity Evaluation in Male Cynomolgus Macaques (Macaca fascicularis)
Source: Animals (Basel). 2026 Aug 19;16(16):2592. doi: 10.3390/ani16162592 (PMC13508964; doi:10.3390/ani16162592)
Supplement: Supplementary file 1 [file animals-16-02592-s001.zip › Supplementary_Figures_histopathology.pdf]

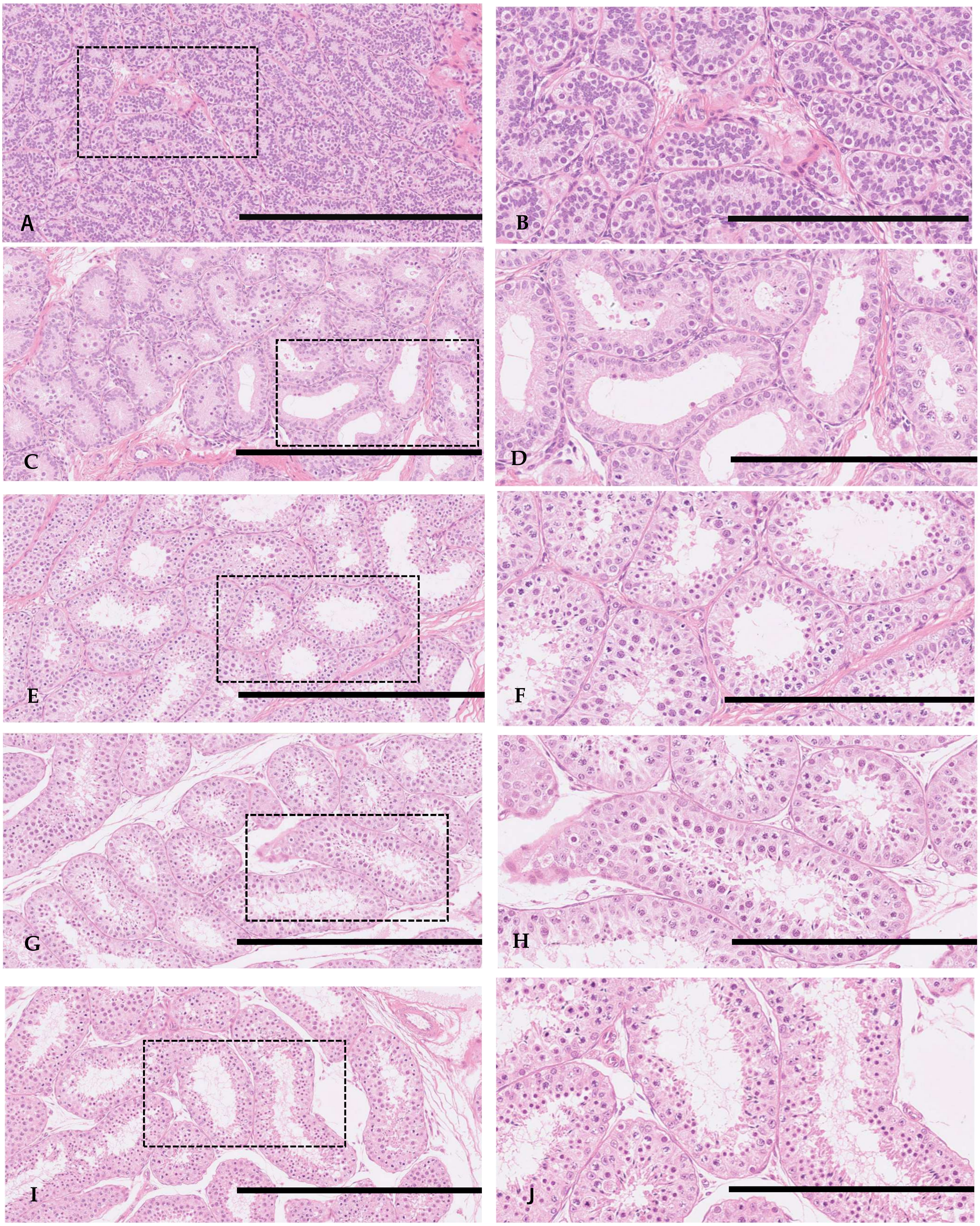
A
B
C
D
E
F
G
H
I
J

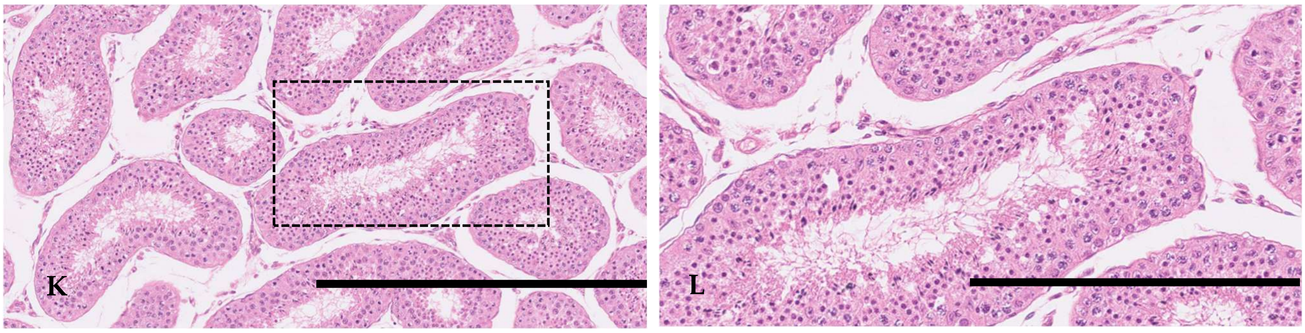

Figure S1. Representative histological features of testes across developmental Grades 1–6 in male cynomolgus macaques. Representative hematoxylin and eosin (H&E)-stained sections of testes classified according to the six-grade histological system described by Haruyama et al. [2]. (A,B) Grade 1 (immature), characterized by seminiferous tubules containing predominantly Sertoli cells and spermatogonia without lumen formation; (C,D) Grade 2 (prepuberty), showing early lumen formation and the appearance of round spermatids but no elongated spermatids; (E,F) Grade 3 (onset of puberty), showing all major germ-cell classes, including elongated spermatids, with relatively small seminiferous tubules and immature spermatogenic organization; (G,H) Grade 4 (puberty), characterized by almost complete spermatogenesis with identifiable spermatogenic stages, although seminiferous tubules remain relatively small; (I,J) Grade 5 (early adult), showing complete spermatogenesis; and (K,L) Grade 6 (adult), showing complete spermatogenesis in well-developed seminiferous tubules. Panels B, D, F, H, J, and L show higher-magnification views of the areas indicated by the dashed boxes in panels A, C, E, G, I, and K, respectively. Scale bars = 500 µm in A, C, E, G, I, and K and 250 µm in B, D, F, H, J, and L.

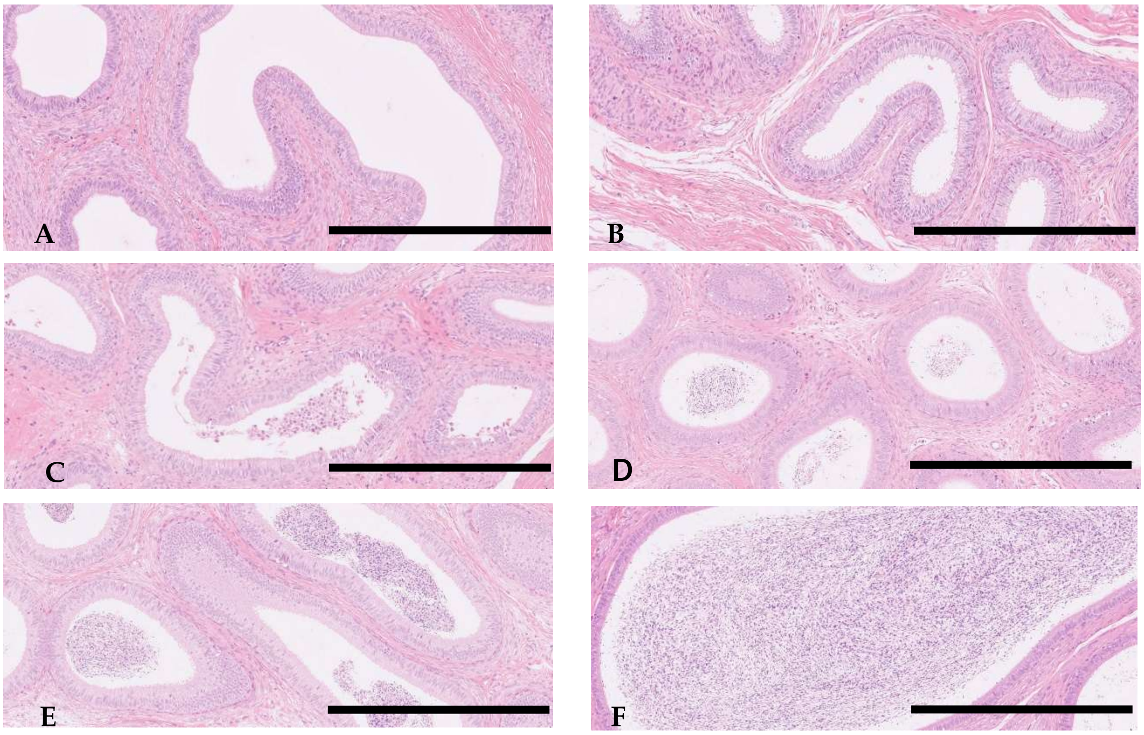

Figure S2. Representative histological features of epididymides across testicular developmental Grades 1–6 in male cynomolgus macaques. Representative hematoxylin and eosin (H&E)-stained sections of epididymides corresponding to the six-grade histological classification described by Haruyama et al. [2]. (A) Grade 1 (immature), showing no sperm and only very slight luminal debris; (B) Grade 2 (prepuberty), showing no sperm and very slight luminal debris; (C) Grade 3 (onset of puberty), showing very few to few sperm and moderate luminal debris; (D) Grade 4 (puberty), showing very few to moderate amounts of sperm with less debris than in Grade 3; (E) Grade 5 (early adult), showing moderate to large amounts of sperm; and (F) Grade 6 (adult), showing large to very large amounts of sperm within the epididymal duct lumen. Scale bars = 500 μm.
