# Supplementary material for: In-Life Testicular Volume as an Indicator of Histological Testicular Development Suitable for Testicular Toxicity Evaluation in Male Cynomolgus Macaques (Macaca fascicularis)
Source: Animals (Basel). 2026 Aug 19;16(16):2592. doi: 10.3390/ani16162592 (PMC13508964; doi:10.3390/ani16162592)
Supplement: Supplementary file 1 [file animals-16-02592-s001.zip › Supplementary_Table_S2_Logistic_Regression_Coefficients.pdf]

Supplementary Table S2. Complete logistic regression coefficients for Models 1–4.

Model	Predictor	β	SE	OR	95% CI for OR	p value	McFadden pseudo-R^2
Model 1	Intercept	-8.3445	2.9773	—	—	0.0051	0.719
Model 1	Mean testicular volume (per 1 cm^3)	0.9886	0.3560	2.69	1.34–5.40	0.0055	0.719
Model 2	Intercept	-12.3705	5.1669	—	—	0.0167	0.735
Model 2	Mean testicular volume (per 1 cm^3)	0.9462	0.3353	2.58	1.34–4.97	0.0048	0.735
Model 2	Age (per 1 year)	0.8451	0.8006	2.33	0.48–11.18	0.2911	0.735
Model 3	Intercept	-22.0628	10.0013	—	—	0.0274	0.764
Model 3	Mean testicular volume (per 1 cm^3)	0.9965	0.3689	2.71	1.31–5.58	0.0069	0.764
Model 3	Body weight (per 1 kg)	2.9220	1.8015	18.58	0.54–634.58	0.1048	0.764
Model 4	Intercept	-23.5655	10.4067	—	—	0.0235	0.771
Model 4	Mean testicular volume (per 1 cm^3)	0.9851	0.3621	2.68	1.32–5.45	0.0065	0.771
Model 4	Age (per 1 year)	0.5904	0.8261	1.80	0.36–9.11	0.4748	0.771
Model 4	Body weight (per 1 kg)	2.6032	1.8174	13.51	0.38–475.88	0.1520	0.771

Notes: Binary logistic regression was performed at the animal level (n = 61). The dependent variable was bilateral histological maturity status, coded as 1 for bilateral Grade ≥4 and 0 for bilateral Grades 1–3. Model 1 included mean testicular volume only; Model 2 included mean testicular volume and age; Model 3 included mean testicular volume and body weight; and Model 4 included mean testicular volume, age, and body weight. β denotes the regression coefficient and SE the standard error. Odds ratios (ORs) and 95% confidence intervals are shown for predictors; ORs are not presented for intercepts. McFadden pseudo-R^2 is reported once per model and repeated across rows for readability.
